# A new type of simulated partial gravity apparatus for rats based on a pully-spring system

**DOI:** 10.3389/fcell.2022.965656

**Published:** 2022-08-31

**Authors:** Shenke Zhang, Takuya Adachi, Shengli Zhang, Yukari Yoshida, Akihisa Takahashi

**Affiliations:** ^1^ Graduate School of Medicine Medical Sciences, Gunma University, Maebashi, Japan; ^2^ Gunma University Heavy Ion Medical Center, Maebashi, Japan

**Keywords:** partial gravity, simulated mechanical stress, bone parpameters, distal femur, proximal tibia

## Abstract

The return to the Moon and the landing on Mars has emphasized the need for greater attention to the effects of partial gravity on human health. Here, we sought to devise a new type of simulated partial gravity apparatus that could more efficiently and accurately provide a partial gravity environment for rat hindlimbs. The new apparatus uses a pulley system and tail suspension to create the simulated partial gravity of the rat’s hind limbs by varying the weight in a balance container attached to the pulley system. An experiment was designed to verify the reliability and stability of the new apparatus. In this experiment, 25 seven-week-old male Wistar Hannover rats were randomly divided into five groups (*n* = 5 per group): hindlimb full weight-bearing control (1*G*), sham (1*G*), and the simulated gravity groups including Mars (3/8*G*), Moon (1/6*G*), and interplanetary space (microgravity: µ*G*). The levels of partial gravity experienced by rat hindlimbs in the Mars and Moon groups were provided by a novel simulated partial gravity device. Changes in bone parameters [overall bone mineral density (BMD), trabecular BMD, cortical BMD, cortical bone thickness, minimum moment of area (MMA), and polar moment of area (PMA)] were evaluated using computed tomography in all rats at the proximal, middle, and distal regions of femur and tibia. Reduced gravity led to decreases in bone parameters (overall BMD, trabecular BMD, cortical BMD, MMA, and PMA) in the simulated gravity groups, mainly in distal femur and proximal tibia. The proximal tibia, MMA, and PMA findings indicated greater weakness in the µ*G* group than in the Mars group. The sham group design also excluded the decrease in lower limb bone parameters caused by the suspension attachment of the rat’s tail. The new simulated partial gravity apparatus can provide a continuous and stable level of partial gravity. It offers a reliable and valuable model for studying the effects of extraterrestrial gravity environments on humans.

## Introduction

The Earth’s unique gravitational environment and atmosphere protect humans from low gravity and various types of radiation. Nevertheless, since the first human mission into space and subsequent landing on the Moon, there has been a need to explore the effects of extraterrestrial environments on human health. This need for exploration has increased with the imminent return to the Moon and the potential for exploration of Mars (NASA 2014, 2020). Current research findings indicate that nearly all systems in the human body will be adversely affected by the altered gravity in extraterrestrial environments; these adverse effects include bone injury (Stein, 2012; Shiba et al., 2017), heart rate alterations (Liu et al., 2015), intracranial hypertension and visual impairment (Zhang and Hargens, 2018), and increased urinary stone risk (Liakopoulos et al., 2012). However, most research has focused on the musculoskeletal system because of its close relationship with gravity (Morey-Holton et al., 2005).

With respect to the effects of extraterrestrial environments on human health, the greatest accuracy can be achieved by collecting data from astronauts and experimental animals in space stations; however, the constraints of launch costs, technology, and physical space on board each spacecraft have prevented such research from becoming widely implemented. Although it is impossible to fully simulate an extraterrestrial environment on Earth, the use of simulators on Earth for analyses of experimental animals is becoming mainstream because of low cost, simplicity, robust control, and generalizability. The benefits of simulators have led to the construction of devices that can simulate low-gravity conditions on Earth. The rat hindlimb unloading methodology established by Morey et al. (1979) has become the standard method used by researchers to simulate extraterrestrial microgravity (µ*G*) on Earth. Numerous studies have been performed using this device, demonstrating its value in analyses of non-weight-bearing hindlimbs on Earth (Morey-Holton et al., 2005).

Nevertheless, as human exploration of space continues, its scope will extend to environments with partial gravity, such as the Moon. Because the rat hindlimb unloading device can only simulate 0 gravity (0*G*), it no longer fully meets experimental requirements. Thus, in 2010, a new device was constructed that could simulate partial gravity in mouse hindlimbs, allowing exposure to partial gravity for extended periods of time during four-limb movement (Wagner et al., 2010). Nonetheless, rats are also often utilized in scientific experiments because of their short interval between litters, high number of pups per litter, adaption to a caged environment, low maintenance costs, docile temperament, and ease of handling (Andersen et al., 2016). In 2018, a partial gravity model for rats was developed. The device achieved the target level of gravity in rats by measuring the body weight ratio and adjusting the chain length during four-limb movement (Mortreux et al., 2018). Its ability to reliably simulate partial gravity was demonstrated by the analysis of skeletal muscles in rats that had been subjected to experiments using the device. However, in this device, using non-retractable stainless-steel chains may limit the range of motion of the rats within the cage. Moreover, the use of attachments at the joints may also impact the range of motion of the joints of the rats. These may be additional factors that cause bone loss. Furthermore, this device requires two rat weight measurements when obtaining a partial gravity level, and too many weight measurements may cause additional stress to the rat.

In this study, we sought to devise a new type of simulated partial gravity apparatus (NA) for the hindlimbs of rats that could more efficiently and accurately simulate a partial gravity environment for rat hindlimbs, which will also reduce the impact of additional factors on the experiment results such as range of motion and number of weight measurements. We used the NA to simulate partial gravity, then assessed its reliability. Our new device may facilitate future utilization and refinement of partial gravity simulation devices.

## Materials and methods

### Animal care

All experimental animals were procured, maintained, and used in accordance with the Recommendations for Handling of Laboratory Animals for Biomedical Research, approved by the Animal Care and Experimentation Committee of Gunma University, Showa Campus (No. 21-081). Twenty-five 7-week-old male Wistar Hannover rats were obtained from Japan SLC, Inc. (Shizuoka, Japan). For acclimation, each rat was individually housed for the first week after acquisition in a room with a temperature of 22 ± 2°C and a 12-h day/night cycle. Food and water were provided *ad libitum*. During the 10-days experiment, new food was provided, and consumption was measured daily for each group of rats; the water was changed at 2-days intervals. Furthermore, bedding in each specialized cage (W 50.5 cm × D 37.0 cm × H 31.5 cm, #82219326, Ryohin Keikaku Co., Ltd., Tokyo, Japan) was changed at 2-days intervals, and the specialized cage was wiped with 70% alcohol during each bedding change. At the end of the 10-days experiment, all rats were euthanized by intraperitoneal injection of 5% sodium pentobarbital.

### Experimental design

**FIGURE 1:**
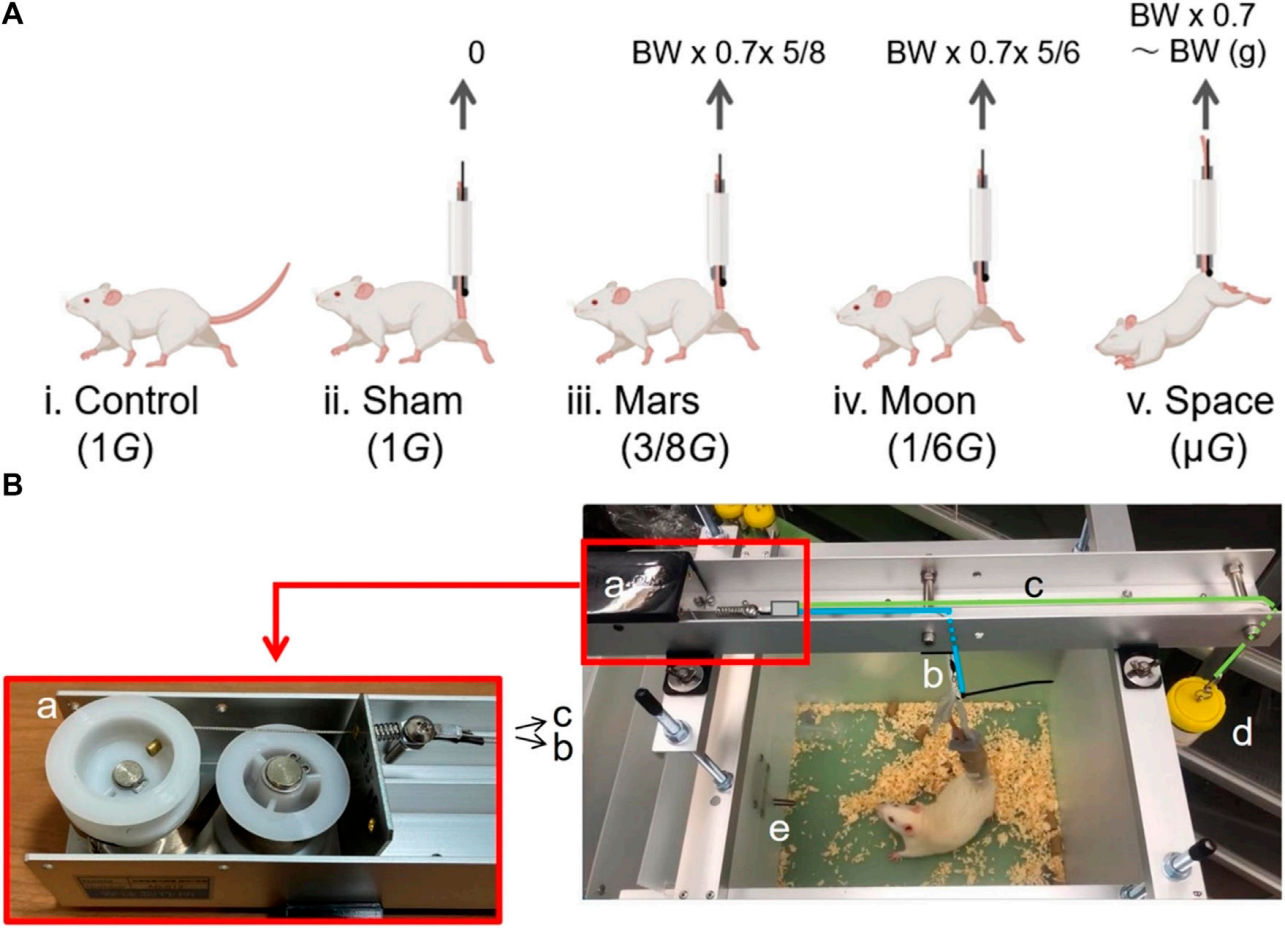
The groups of experimental rats **(A)** and the core components of the new type of simulated partial gravity apparatus **(B)**. Control group (1*G*) **(Ai)**; sham group with SA (1*G*) **(Aii)**; Mars group (3/8*G*) **(Aiii)**; Moon group (1/6*G*) **(Aiv)**; and interplanetary space (µ*G*) group **(Av)**. Black spring storage box with two built-in pulleys and a spring wire splitter **(Ba)**; spring wire connected to the rat’s tail **(Bb)**; spring wire connected to the balance container **(Bc)**; balance container **(Bd)**; and partition **(Be)**.

On the last day of the acclimation period, rats were randomly assigned to one of five groups (*n* = 5 per group): control, (i.e., Earth gravity, with hindlimb weight of 1*G*; Figure 1Ai); sham (hindlimb weight of 1*G*, plus tail suspension; Figure 1Aii); Mars (hindlimb weight of 3/8*G*; Figure 1Aiii); Moon (hindlimb weight of 1/6*G*; Figure 1Aiv); and µ*G*; Figure 1Av). As described in other surveys, the rats in each group were subjected to hindlimb suspension for 10 days (Kawano et al., 2004; Ohira et al., 2011; Zhang et al., 2021).

On the first day of the experiment, the five groups were treated differently depending on the level of gravity as follows. Rats in the control group were transferred to a new cage with specifications identical to the cage used during the acclimation period. Rats in the Mars and Moon groups were transferred to a specialized cage equipped with the NA; their tails were attached to the suspension apparatus to receive 10 days of simulated Mars and Moon gravity levels. Rats in the sham and µ*G* groups were transferred to the specialized cages used for the Mars and Moon groups. However, the cages used the normal lid instead of the NA. The tails of both groups of rats were attached to the lid by a plastic rope, and the hindlimbs were subjected to Earth and outer space gravity levels for 10 days.

The hindlimbs of rats in the sham group remained fully in contact with the cage bottom, while the hindlimbs of rats in the µ*G* group were not allowed to contact the cage bottom. Rats in the µ*G* group relied solely on their forelimbs to move and eat inside the specialized cage. The angle between each rat’s torso and the bottom of the cage was approximately 30°. However, the rats in the simulated Mars and Moon gravity groups, the linkage of the tail to NA allows the use of the four limbs to move freely within the box, avoiding the creation of an angle between the head and the bottom of the box and further avoiding the redistribution of fluid throughout the body.

To ensure that the rats in the sham group did not escape the suspension, their tails were re-suspended every 3 days during the experiment. The tail suspension status of each rat’s tail was checked every morning and afternoon; any rats that escaped the suspension apparatus were excluded from further analysis.

### New partial gravity simulation apparatus

The NA was designed with particular attention to stability, reliability, and convenience. In terms of stability, the simulator must provide a continuous and stable partial gravity environment for rat hindlimbs. Therefore, we ensured that partial gravity fluctuation was controlled within a specific range to avoid the introduction of bias into experimental results. Concerning reliability, the simulator must accurately simulate the target level of gravity to ensure optimal weight-bearing for rat hindlimbs during the experiment. Finally, in terms of convenience, the simulator must be simple, and easily used by experimental personnel; these characteristics can facilitate modification and dissemination if the simulator meets experimental requirements.

### Composition of the new partial gravity simulation apparatus

The NA comprises four stainless-steel brackets and a long stainless-steel slot; the brackets can be attached to a specialized cage. In the upper bracket, a fixed stainless-steel slot contains the core of the NA. The core consists of five parts: a black spring storage box with two built-in pulleys and a spring wire splitter (Figure 1Ba); a spring wire connected to the rat’s tail (Figure 1Bb); a spring wire connected to the balance container (Figure 1Bc); a balance container to adjust the force on the rat’s hindlimbs (Figure 1Bd); and a partition that separates the water bottle and the rat’s activity space (Figure 1Be).

From the left side to the right side of the long slot, the spring storage box and the spring wire splitter are connected but movable; the upper section of the spring wire (Figure 1Bb) is connected to one end of the spring splitter (Figure 1Ba), while the lower section is connected to the rat’s tail by a removable swivel button. The upper section of the spring wire c (Figure 1Bc) is connected to the other side of the spring splitter (Figure 1Ba), while a swivel button is attached to the lower end on the right side; the upper end of the lid of the balance container (Figure 1Bd) is attached to a swivel button, which can be connected to the swivel button of the right lower section (Figure 1Bc). To prevent the rat from climbing onto the water bottle, the water bottle is placed in a separate space inside the specialized cage; the outlet of the water bottle enters the rat’s activity space inside the specialized cage through a small hole (Figure 1Be).

### Operation of the novel partial gravity simulation apparatus

Through the two pulleys inside, the spring splitter moves away from the black spring storage box (toward the right side) when pulled by spring wires (Figures 1Bb,Bc). Two polyurethane foam adhesive sponges (W 30 mm × t 10 mm, #E0230, Nitomos Inc., Tokyo, Japan), a non-stretchable taping tape (W 12 mm, #CH-12, Nitiban Co., Ltd., Tokyo, Japan), and a plastic (polypropylene) rope (W 60 mm, #B174J, Jointex., Tokyo, Japan) are wrapped around the rat’s tail and secured; the top end of the plastic rope is connected to the swivel button shown in Figure 1Bb, ensuring that the rat’s hindlimbs are separated from the bottom of the specialized cage. At this point, the rat’s hindlimbs cannot make contact with the bottom of the specialized cage, although they are near the bottom. The gravitational force on each rat’s hindlimbs is completely counteracted by the upward elastic force generated by the spring wire (Figure 1Bb). Each spring system has its own unique and constant elastic force, which can be partially offset by the tension force generated by the balance container (Figure 1Bd). When the swivel button of the wire in Figure 1Bc is connected to the balance container (Figure 1Bd), the tension generated by the balance container moves the spring splitter (Figure 1Ba) to the right. At this point, both the swivel button of the wire in Figure 1Bb and the right end of the wire in Figure 1Bc are lowered; the rat’s hindlimbs descend to the bottom of specialized cage, allowing the rat to use all four limbs to eat and drink while maintaining the expected level of partial gravity.

In this study, the following calculations were used to determine the weight of the balance container (Figure 1Bd). For simulation of partial gravity on the Moon: balance container weight (BCW) = spring force (SF)–0.7 × daily weight (DW) × 5/6 (0.7 × DW–0.7 × DW × 1/6). For simulation of partial gravity on Mars: BCW = SF–0.7 × DW × 5/8 (0.7 × DW–0.7 × DW × 3/8). After determination of the rat DW, the BCW was calculated *de novo* using the corresponding formula above to ensure that the rat hindlimb weight was always subjected to the desired level of gravity through the addition or removal of lead weight blocks in the balance container. Using a method that expressed paw pressure as a percentage of total body weight, Pertici et al. (2014) showed that rats exerted a pressure of 15% in each of the left and right forepaws, and a pressure of 35% in each of the left and right hind paws. Therefore, because the rat tail suspension mainly affected each rat’s hindlimbs, we determined the BCW by using 70% of the total weight as the basis for calculation.

### Weight assessment

The rats in each group were weighed daily. Depending on the group. We used three (i, ii, and iii) different ways to obtain the daily weight of each group of rats.

(i) We directly obtained the control rats’ body weight by placing them on the scale each day.The weight assessment of the four tail suspension groups was performed as follows. On the morning of the first day of the experiment, the rats were placed on a table. Their tails were grasped to produce a state of stress that led to considerable excretion of feces and urine; rats were weighed after this excretion to obtain more accurate weight values. After a rat had undergone weight assessment, its tail was wrapped with plastic rope, adhesive sponge, and tape. The adhesive side of the sponge with the acrylic adhesive was in direct contact with the tail of the rat.

(ii) For the simulated Mars and Moon gravity level groups, the upper end of the plastic rope was connected to the lower end of the NA (Figure 1Bb) *via* ligation to the swivel button. The suspension attachments (SA) which contains four items (the swivel button, adhesive sponge, plastic rope, and tape) and rat were then removed from the apparatus: the total weight of the SA and body weight was recorded. The SA weight was obtained through subtraction of the body weight that had been previously acquired. On days 2–10 of the experiment, weight assessments were performed as follows. The swivel button at the lower end of the apparatus in Figure 1B was unfastened, allowing the rat and SA to be separated from the apparatus. The total weight of the SA and rat was measured; the DW was obtained through subtraction of SA weight. Each rat was suspended in a box above the scale to calculate the total weight of the SA and rat. Additionally, the hindlimbs of rats in the Mars and Moon groups were allowed to float for a very short period without stress to avoid the entire weight-bearing in hindlimbs during weight assessment.

(iii) For the sham and simulated µ*G* groups, the upper end of the plastic rope was directly connected to the lid. The total weight of the rat, SA (without the swivel button), and lid was measured; the total weight of SA (without the swivel button) and lid was obtained through subtraction of the rat weight that had been previously acquired. On days 2–10 of the experiment, weight assessments were performed as follows. The total weight of the rat, SA (without the swivel button), and lid was measured; the DW was obtained through subtraction of the total SA (without the swivel button) and lid weight. Notably, when measuring the total weight of the rat, SA (without the swivel button), and lid, the hindlimbs of rats in the sham group remained fully in contact with the cage bottom during the assessment; the hindlimbs of rats in the µ*G* group were not allowed to contact the cage bottom.

### Bone sampling and analyses

All rats were dissected; their femurs and tibias were removed for analysis. The bones were cleaned by immersion in 5% papain at 50°C for 24 h. The length of each femur and tibia were recorded. The femur and tibia were divided into three regions (proximal, middle, and distal) using a previously described method (Zhang et al., 2021). The bone parameters of each region were also evaluated: overall bone mineral density (BMD), trabecular BMD, cortical BMD, cortical bone thickness, and mechanical indexes [minimum moment of area (MMA), and polar moment of area (PMA)]. Computed tomography (CT) scans (Latheta LCT-200, Hitachi, Ltd., Tokyo, Japan) were also used to quantify the volume of each femur and tibia as well as the trabecular and cortical area percentage in the femur distal and tibia proximal. The configuration was a voxel size of 48 μm × 96 μm × 96 μm at a tube voltage of 50 kVp, and a tube current of 0.5 mA.

### Statistical analysis

GraphPad Prism 9 software (GraphPad Software Inc., San Diego, CA, United States) was used for data analysis. Shapiro-Wilk normality test and kolmogorow-Smirnov normality test Were used for normality test. All data which passed the normality test are expressed as means ± SD. Significant differences among groups (control, sham, Mars, Moon, and µ*G*) were identified using one-way analysis of variance, followed by post hoc Tukey tests. All data which didn’t passed the normality test are expressed by median (P25, P75). Non-parametric tests (kruskal-wallis test) were used for non-normal distributions. The threshold for statistical significance was regarded as *p* < 0.05.

## Results

### Food intake and weight change

**FIGURE 2:**
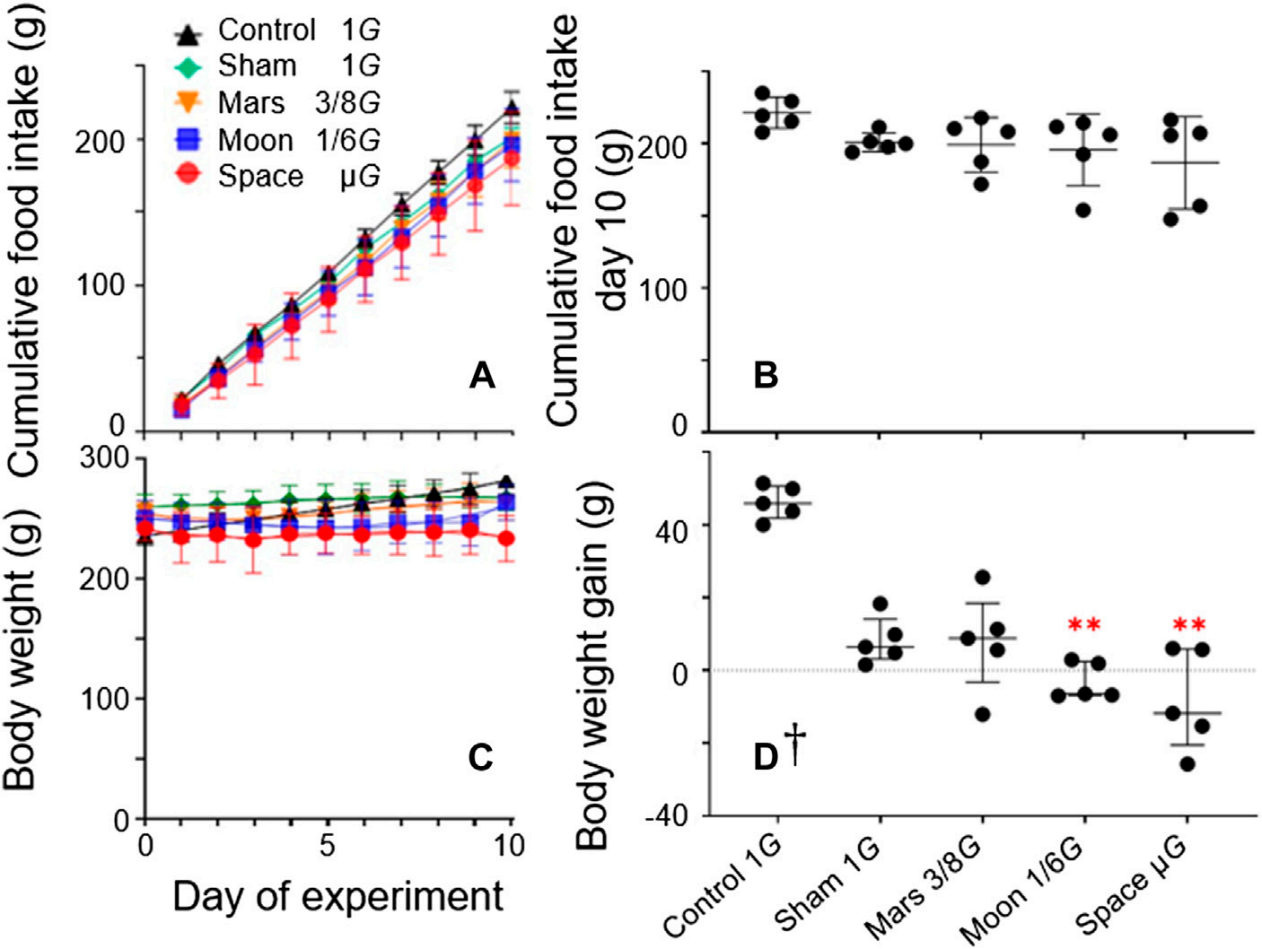
Stress exerted by suspension was measured by cumulative food intake during the experiment period **(A)** and cumulative food intake on day 10 **(B)**; daily body weight during the experiment **(C)** and overall weight gain measured at the end of the experiment **(D)** [Symbols in **(C)** are identical to symbols in **(A)**. Mean ± SD, one-way analysis of variance; † M (P25, P75), kruskal-wallis test, *n* = 5. **p < 0.01 vs. control group].

Capacity to adapt to the experimental environment was evaluated based on changes in the rats’ food consumption and body weight during the experiment (Figure 2). Total food consumption did not significantly differ among the five groups (Figures 2A,B). The body weights of rats in the Mars and

µ*G* groups decreased during the first 3 days of the experiment, then plateaued. The body weight of rats in the Moon group decreased during the first 5 days of the experiment, then plateaued. During the experiment, the body weights of rats in both the control and sham groups significantly increased from baseline; the rate of increase was significantly greater in the control group (+19.1%, *p* < 0.0001; and +3.0%, *p* < 0.05) (Figure 2C). In contrast, the body weights of rats in the simulated gravity groups did not significantly differ from baseline. At the end of the experiment, the final body weights of rats in the Moon and µ*G* groups were significantly lower the final body weight of rats in the control group (−11.8%, *p* < 0.01; −16.7%, *p* < 0.001) (Figure 2C), whereas the body weights of rats in the sham and Mars groups did not significantly differ from the body weight of rats in the control group. Furthermore, weight changes in the Moon and µ*G* groups significantly differed from the weight change in the control group (−106.7% and −117.7%; *p* < 0.001). However, weight changes in the Mars, Moon, and µ*G* groups did not significantly differ from the weight change in the sham group. Moreover, weight changes did not significantly differ among the Mars, Moon, and µ*G* groups (Figure 2D).

### Morphological changes in the femur and tibia

**FIGURE 3:**
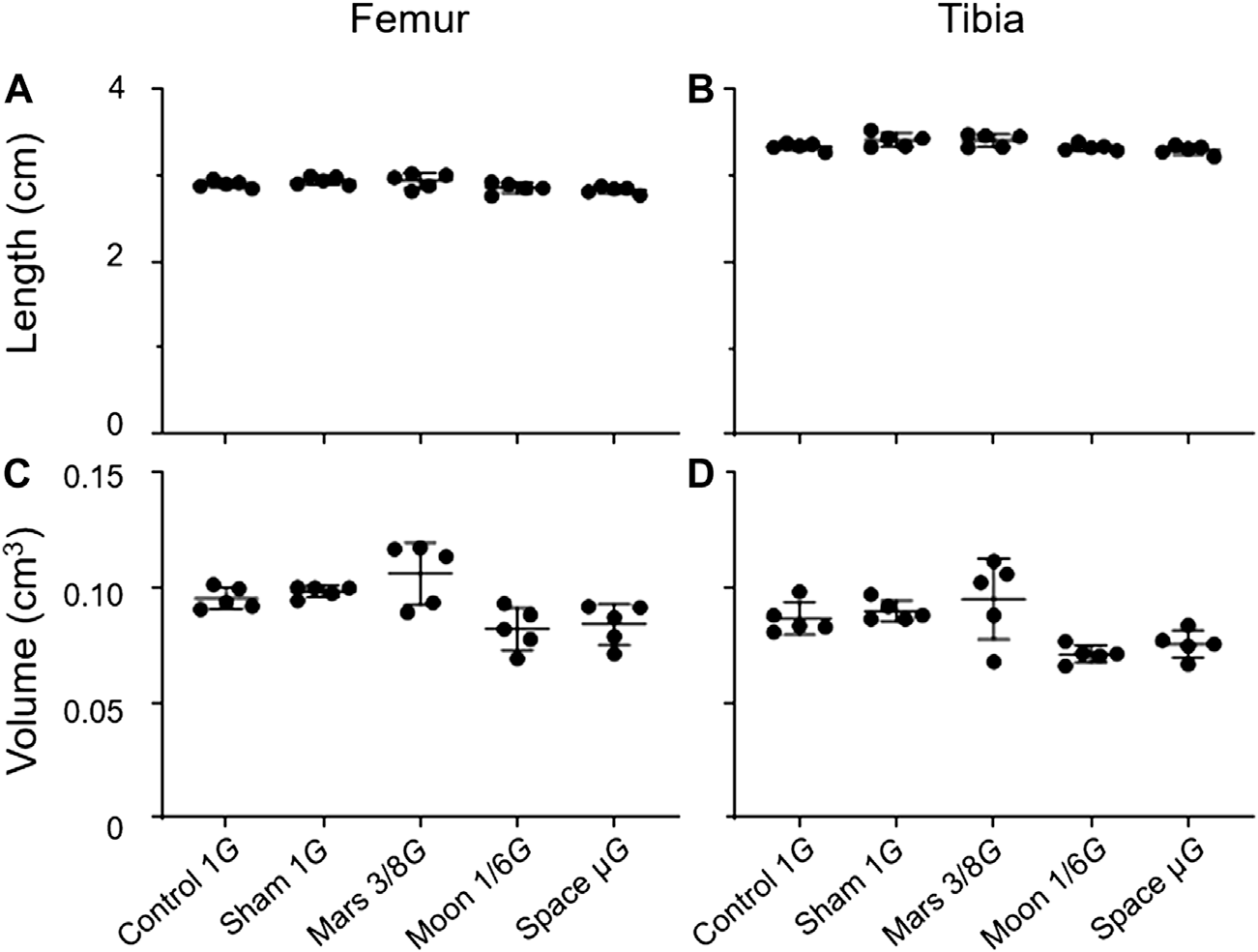
Lengths and volumes of the femur and tibia. **(A)** Femoral length, **(B)** tibial length, **(C)** femoral volume, and **(D)** tibial volume (Mean ± SD, one- way analysis of variance).

To determine the impact of the simulated gravity on bone morphology in rat hindlimbs, we compared femur and tibia lengths and volumes among groups (Figure 3). We found no significant differences among the simulated groups, or between the simulated gravity groups and the control group.

**FIGURE 4:**
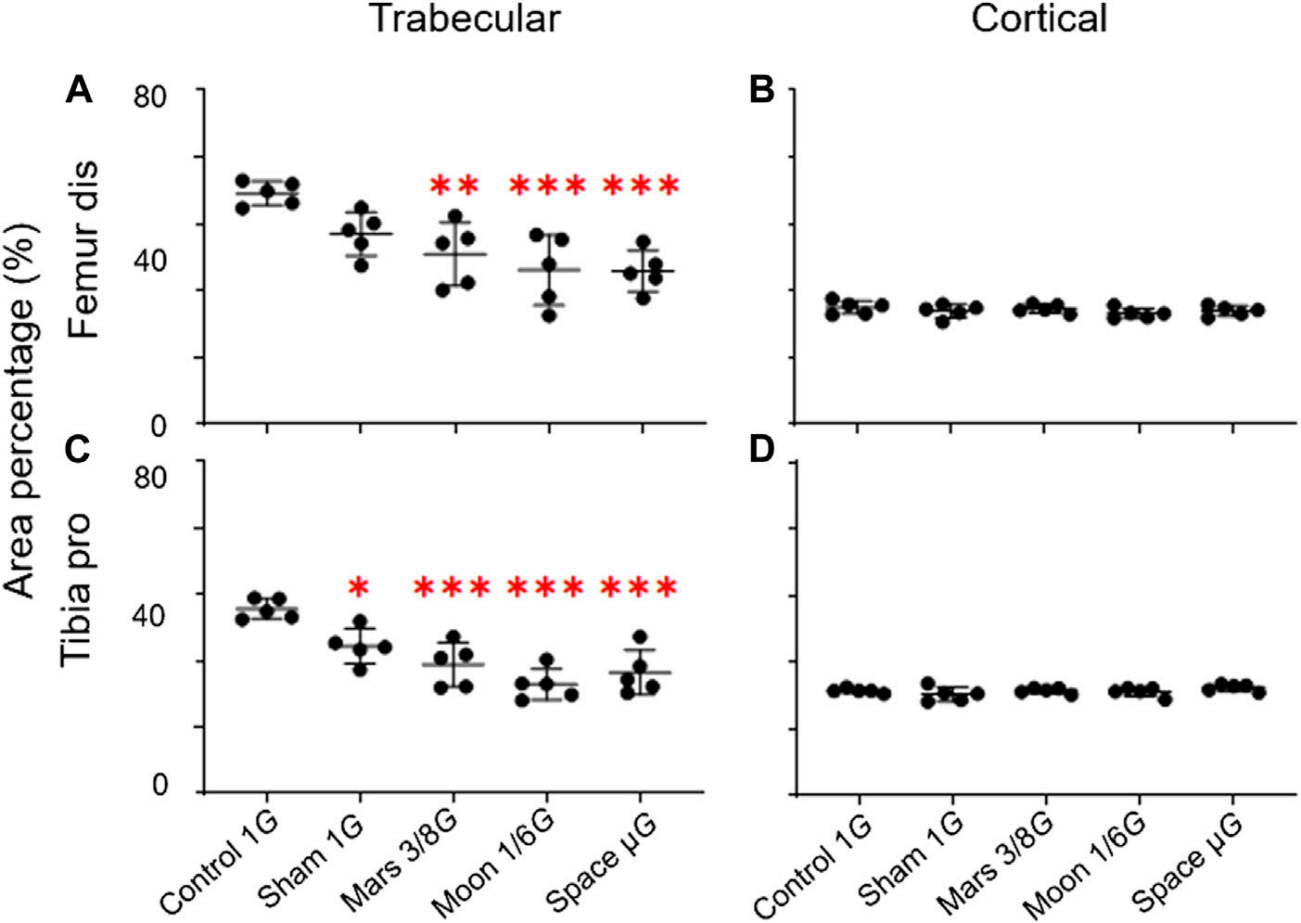
Area percentage of the trabecular and cortical bone in the distal femur and proximal tibia. **(A)** Trabecular area percentage in the distal femur, **(B)** cortical area percentage in the distal femur, **(C)** trabecular area percentage in the proximal tibia, and **(D)** cortical area percentage in the proximal (mean ± SD, *p < 0.05 vs. control group, **p < 0.01 vs. control group, ***p < 0.001 vs. control group, one-way analysis of variance).

The trabecular and cortical area percentage in the distal femur and proximal tibia were also observed. No significant difference in the cortical area percentage of the simulated gravity groups compared with that of the control group at the distal femur and proximal tibia was observed. However, for the trabecular area percentage, the simulated Mars, Moon, and µ*G* groups at the distal femur decreased significantly compared with the control group (−26.3%, *p* < 0.01; −33.2%, *p* < 0.001; and −33.7%, *p* < 0.001) (Figure 4A), and the sham, simulated Mars, Moon, and µ*G* groups at the proximal tibia had similar decreases (−20.1%, *p* < 0.05; −30.2%, *p* < 0.001; −40.9%, *p* < 0.001; and −34.3%, *p* < 0.001) (Figure 4C).

### Changes in femoral bone parameters

**FIGURE 5:**
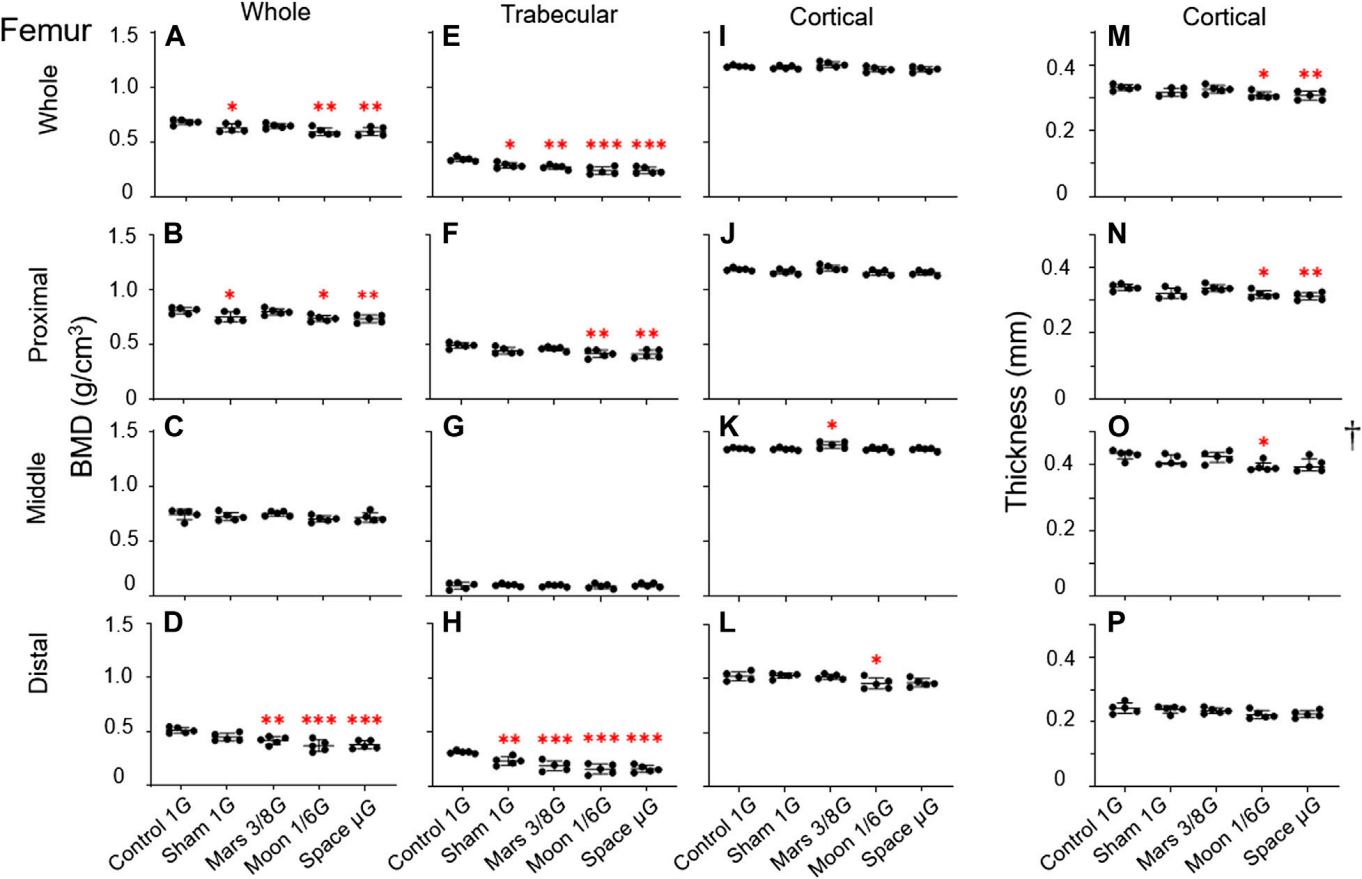
Bone parameters of various femoral regions compared among the five experimental groups. **(A–D)** Overall BMD, **(E–H)** trabecular BMD, **(I–L)** cortical BMD, and **(M–P)** cortical bone thickness. **(A,E,I,M)** Whole femur, **(B,F,J,N)** proximal femur, **(C,G,K,O)** middle femur, and **(D,H,L,P)** distal femur [Mean ± SD, one-way analysis of variance; †M (P25, P75), kruskal-wallis test, *n* = 5. *p < 0.05 vs. control group, **p < 0.01 vs. control group, ***p < 0.001 vs. control group].

Overall BMD: Analyses of overall BMD revealed statistically significant differences (*p* < 0.01, *p* < 0.01, and *p* < 0.001) among the five groups in whole femur, as well as proximal and distal regions (Figures 5A,B,D). The greatest decrease in overall BMD was observed in the distal region in the simulated gravity groups, such that it was lower in the Mars, Moon, and µ*G* groups than in the control group (−18.4%, *p* < 0.01; −27.8%, *p* < 0.001; and −26.2%, *p* < 0.001) (Figure 5D). Compared with the sham group, the Moon and µ*G* groups exhibited overall BMD reductions of −17.6% and −15.7% (*p* < 0.05 and *p* < 0.05), respectively, in distal femur. However, overall BMD in distal femur did not significantly differ among the Mars, Moon, and µ*G* groups. Furthermore, overall BMD in whole femur was significantly lower in the sham, Moon, and µ*G* groups than in the control group (−7.9%, *p* < 0.05; −13.1%, *p* < 0.01; and −12.6%, *p* < 0.01) (Figure 5A). Similarly, overall BMD in proximal femur was significantly lower in the sham, Moon, and µ*G* groups than in the control group (−6.9%, *p* < 0.05, −8.8%, *p* < 0.05 and −9.2%, *p* < 0.01) (Figure 5B). However, overall BMD in middle femur did not significantly differ among groups (Figure 5C).

Trabecular BMD: The greatest simulated gravity-induced decline in trabecular BMD was found in distal femur (*p* < 0.0001) (Figure 5H). In the distal section, the sham, Mars, Moon, and µ*G* groups all exhibited significantly lower trabecular BMD, compared with the control group (−25.9%, *p* < 0.01; −40.2%, *p* < 0.001; −49.9%, *p* < 0.001 and −49.1%, *p* < 0.001) (Figure 5H). Trabecular BMD was significantly lower in the Moon and µ*G* groups than in the sham group (−32.5%, *p* < 0.05 and −31.4%, *p* < 0.05). However, trabecular BMD did not significantly differ among the three simulated gravity groups. Similar to overall BMD, the experimental groups exhibited various extents of trabecular BMD reduction in whole and proximal femur, compared with the control group. Notably, the sham, Mars, Moon, and µ*G* groups had distinct degrees of trabecular BMD reduction in whole femur (−16.3%, *p* < 0.05; −21.0%, *p* < 0.01; −30.4%, *p* < 0.001 and −29.9%, *p* < 0.001) (Figure 5E).

The Moon and µ*G* groups also exhibited substantial trabecular BMD reduction in proximal femur (−15.6%, *p* < 0.01 and −16.1%, *p* < 0.01) (Figure 5F), but the reduction was smaller than in distal and whole femur. Finally, no trabecular BMD reductions in middle femur were found among the simulated gravity groups (Figure 5G).

Cortical BMD: The changes of cortical BMD were also observed in this study. Cortical BMD in middle femur was significantly higher in the Mars group than in the control group (+2.5%, *p* < 0.05) (Figure 5K). Cortical BMD of the Moon group in distal femur was significantly lower than in the control group (−6.4%, *p* < 0.05) (Figure 5L). No significant differences in cortical BMD were found in other regions of the femur.

Cortical bone thickness: The results of cortical bone thickness analyses were inconsistent with other bone parameter findings. With the exception of distal femur, we observed varying degrees of cortical bone thickness reduction in whole femur (−7.0%, *p* < 0.05 and −7.3%, *p* < 0.01) (Figure 5M) and proximal femur (−6.3%, *p* < 0.05 and −8.0%, *p* < 0.01) (Figure 5N) in both the Moon and µ*G* groups after comparing the control group. Additionally, the decrease in cortical thickness in the simulated Moon group in the middle femur was less than that in the control group (−8.3%, *p* <0.01) (Figure 5O).

### Changes in tibial bone parameters

**FIGURE 6:**
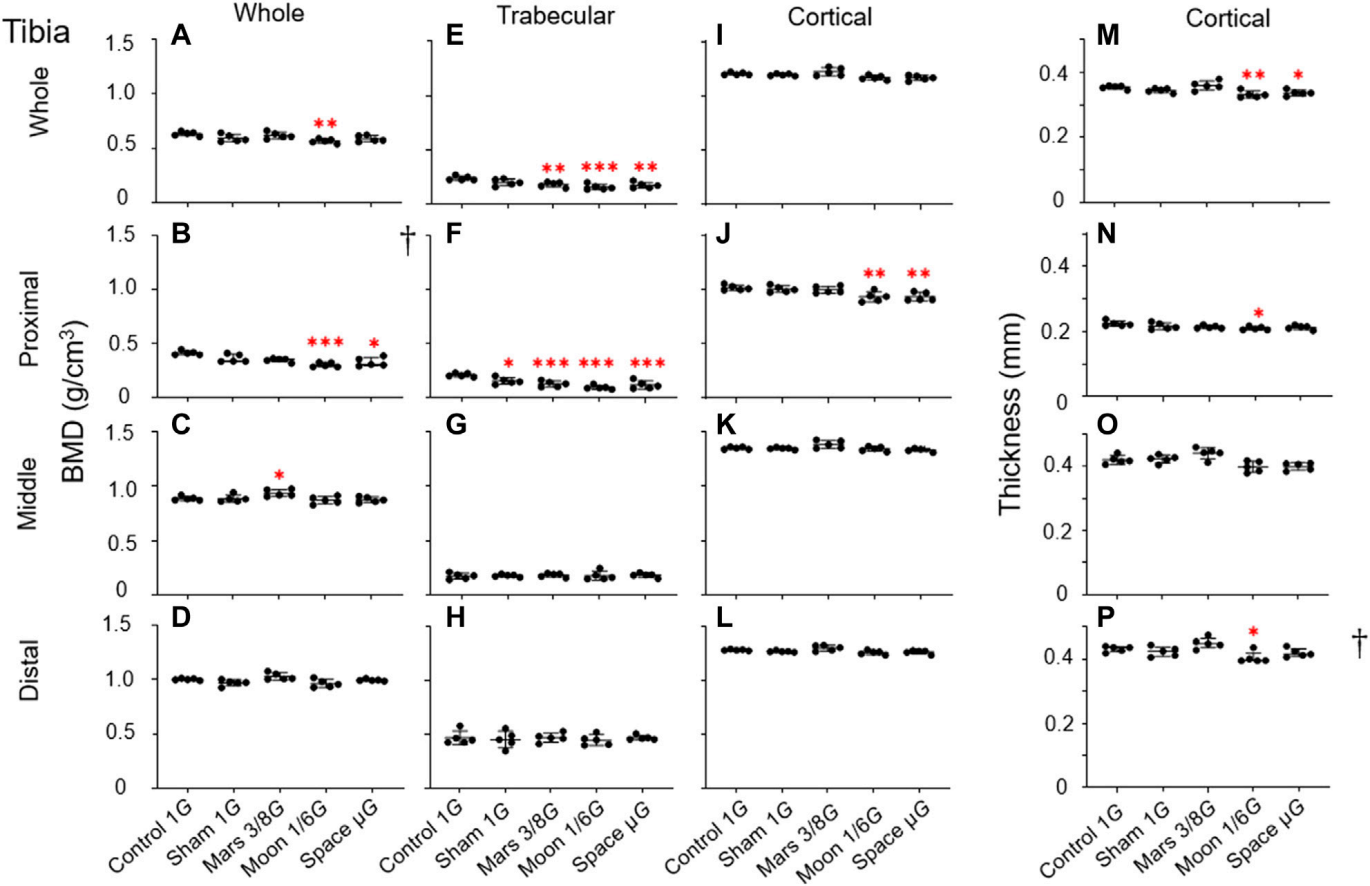
Bone parameters of various tibial regions compared among the five experimental groups. **(A–D)** Overall BMD, **(E–H)** trabecular BMD, **(I–L)** cortical BMD, and **(M–P)** cortical bone thickness. **(A,E,I,M)** Whole tibia, **(B,F,J,N)** proximal tibia, **(C,G,K,O)** middle tibia, and **(D,H,L,P)** distal tibia [Mean ± SD, one-way analysis of variance; † M (P25, P75), kruskal-wallis test, *n* = 5. *p < 0.05 vs. control group, **p < 0.01 vs. control group, ***p < 0.001 vs. control group].

Overall BMD: Decreases in overall BMD in the simulated gravity groups were mainly observed in proximal tibia. In this area, the Moon and µ*G* groups had significantly lower overall BMD, compared with the control group (−26.8%, *p* < 0.001 and −20.6%, *p* < 0.05) (Figure 6B). Only the Moon group exhibited significantly lower overall BMD in this region, compared with the sham group (−16.5%, *p* < 0.05); there were no significant differences among the three simulated gravity groups. Moreover, the Moon group exhibited significantly lower overall BMD in whole tibia compared to the control group (−10.6%, *p* < 0.01) (Figure 6A). Surprisingly, overall BMD in middle tibia was greater in the Mars group (+5.6%, *p* < 0.05) than in the control group (Figure 6C).

Trabecular BMD: Similar to the overall BMD, the decline in trabecular BMD in the tibia was mainly observed in the proximal region. In the proximal section, trabecular BMD was lower in the sham group than in the control group (−25.9%, *p* < 0.05); the magnitude of the reduction in trabecular BMD was considerably larger in the Mars, Moon, and µ*G* groups (−39.1%, *p* < 0.001; −54.2%, *p* < 0.001 and −43.6%, *p* < 0.001) (Figure 6F). Furthermore, in this region, only the Moon group exhibited significantly lower trabecular BMD compared with the sham group (−38.2%, *p* < 0.05); there were no significant differences in trabecular BMD among the three simulated gravity groups. Similar to proximal tibia, we found a significant trabecular BMD reduction in whole tibia in the Mars, Moon, and µ*G* groups (−23.9%, *p* < 0.01; −33.9%, *p* < 0.001 and −27.2%, *p* < 0.01), compared with the control group (Figure 6E). In middle and distal tibia, no reductions in trabecular BMD were observed in any of the simulated gravity groups (Figures 6G,H).

Cortical BMD: In contrast to the overall and trabecular BMD findings in tibia, our analysis showed that simulated gravity had a mild effect on cortical bone, which was limited to proximal tibia. Compared with the control group, cortical BMD in proximal tibia was significantly lower in the Moon and µ*G* groups (−8.2% and −8.1%, *p* < 0.01); it was also significantly lower in the µ*G* group than in the Mars group (−6.8%, *p* < 0.05) (Figure 6J). However, no cortical BMD reductions were observed in other tibial regions among the simulated gravity groups (Figures 6I,K,L).

Cortical bone thickness: Reductions of cortical bone thickness were observed in some simulated gravity groups (Figures 6M,N,P). Cortical bone thickness in whole tibia was lower in the Moon and µ*G* groups than in the control group (−6.3%, *p* < 0.01 and −4.9%, *p* < 0.05) (Figure 6M). In contrast, cortical bone thickness in proximal was only lower in the Moon group than in the control group (−6.3%, *p* < 0.05) (Figure 6N). No reductions of cortical bone thickness in middle and distal tibia were observed in any simulated gravity group (Figure 6O).

### Mechanical indexes of the femur and tibia

To observe the effects of changes in gravity on femoral and tibial mechanics, we analyzed mechanical indexes of the femur and tibia.

**FIGURE 7:**
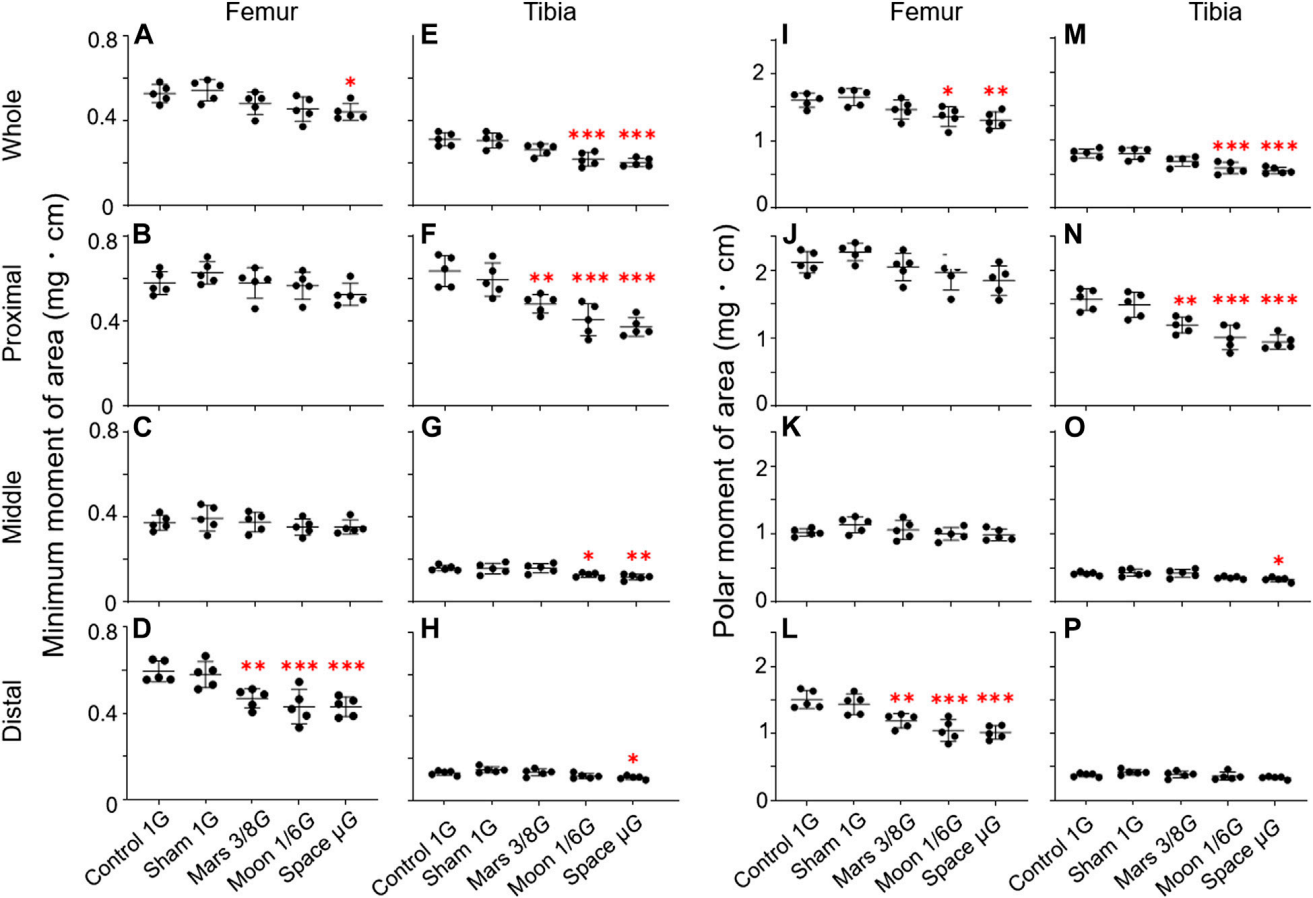
Mechanical indexes of the femur and tibia [minimum moment of inertia of area (MMA) and polar moment of inertia of area (PMA)] compared among the five experimental groups. **(A–D)** Femoral MMA, **(E–H)** tibial MMA, **(I–L)** femoral PMA, and **(M–P)** tibial PMA. **(A,E,I,M)** Whole femur/tibia, **(B,F,J,N)** proximal femur/tibia, **(C,G,K,O)** middle femur/tibia, and **(D,H,L,P)** distal femur/tibia (Mean ± SD, *n* = 5. *p < 0.05 vs. control group, **p < 0.01 vs. control group, ***p < 0.001 vs. control group, one-way analysis of variance).

MMA: MMA was significantly lower in the simulated gravity groups, primarily in distal femur and proximal tibia (Figures 7D,F). In distal femur, MMA was significantly lower in the Mars, Moon, and µ*G* groups when comparing to the control group (−21.1%, *p* < 0.01; −27.5%, *p* < 0.001; and −27.6%, *p* < 0.001) (Figure 7D). It was also significantly lower in the Mars, Moon, and µ*G* groups than in the sham group (−19.2%, *p* < 0.05; −25.8%, *p* < 0.01; and −25.8%, *p* < 0.01). However, no significant differences in MMA were found among the three simulated gravity groups. In proximal tibia, MMA was significantly lower in the Mars, Moon, and µ*G* groups compared to the control group (−24.2%, *p* < 0.01; −36.0%, *p* < 0.001 and −41.3%, *p* < 0.001). In this region, MMA was also significantly lower in the Mars, Moon, and µ*G* groups, compared with the sham group (−19.2%, *p* < 0.05; −31.7%, *p* < 0.001; and −37.4%, *p* < 0.001). In contrast to distal femur, we found that MMA in proximal tibia was significantly lower in the µ*G* group than in the Mars group (−29.1%, *p* < 0.05) (Figure 7F). In other regions of the femur and tibia, the simulated gravity groups also exhibited MMA reductions. In whole femur, MMA was significantly lower in the µ*G* group than in the control group (−16.3%, *p* < 0.05) (Figure 7A); It was also significantly lower in the Moon and µ*G* groups in the whole tibia section compared to the control group (−29.8%, *p* < 0.001 and −34.6%, *p* < 0.001) (Figure 7E). In middle tibia, the reductions of MMA in the Moon and µ*G* groups were −20.6% and −26.5%, respectively (*p* < 0.05 and *p* < 0.01) (Figure 7G); in distal tibia, the reduction of MMA in the µ*G* group was −17.0% (*p* < 0.05) (Figure 7H).

PMA: Similar to MMA, we found that alterations in PMA were concentrated in distal femur and proximal tibia. In distal femur, PMA was significantly lower in the Mars, Moon, and µ*G* groups than in the control group (−21.0%, *p* < 0.01; −30.6%, *p* < 0.001 and −32.5%, *p* < 0.001) (Figure 7L). It was also noticeably lower in the Mars, Moon, and µ*G* groups than in the sham group (−17.3%, *p* < 0.05; −27.4%, *p* < 0.001 and −29.4%, *p* < 0.001). However, no significant differences in PMA were found among the three simulated gravity groups. In proximal tibia, PMA was significantly lower in the Mars, Moon, and µ*G* groups than in the control group (−23.9%, *p* < 0.01; −35.5%, *p* < 0.001 and −39.8%, *p* < 0.001). It was also noticeably lower in the Mars, Moon, and µ*G* groups than in the sham group (−19.9%, *p* < 0.05; −32.2%, *p* < 0.001 and −36.7%, *p* < 0.001); furthermore, PMA was significantly lower in the µ*G* group than in the Mars group (−26.5%, *p* < 0.01) (Figure 7N). In other regions of the femur and tibia, the simulated gravity groups also exhibited PMA reductions. In whole femur, PMA was lower in the Moon and µ*G* groups than in the control group (−15.2%, *p* < 0.05 and −18.5%, *p* < 0.01) (Figure 7I); it was also lower in whole tibia in these groups, compared with the control group (−15.2%, *p* < 0.05 and −18.5%, *p* < 0.01) (Figure 7M). In middle tibia, PMA was lower in the µ*G* group than in the control group (−20.7%, *p* < 0.05) (Figure 7O).

## Discussion

In this study, we devised and tested an NA for rats based on a pully-spring system. The apparatus uses a unique pulley-spring system to ensure that a rat’s hindlimbs experience a specific weight-bearing level by varying the weight in a balance container. We assessed the performance of the NA in terms of food intake, body weight, bone morphological changes, and multiple rat hindlimb bone parameters.

Food intake has been widely used in experiments with rats as an indicator that can assess potential acute or chronic stress. In rats with non-weight-bearing hindlimbs, additional pressure will activate the hypothalamic-pituitary-adrenocortical system and cause an increase in corticosterone levels (Wronski et al., 1998; Chapes et al., 1999), which partially counteracts the effects of altered gravity (Globus and Morey-Holton, 2016). In the present study, the cumulative food intakes of the sham group and the three simulated gravity groups did not significantly differ from the cumulative food intake of the control group. Furthermore, cumulative food intake did not significantly differ among the three simulated gravity groups (Figure 2B). These results suggest that the NA did not cause additional stress to rats during our experiments. Thus, the changes in rat hindlimb bone parameters were primarily caused by the simulated gravity reduction induced by the NA. Notably, the first few days of the experimental period, rats in the simulated gravity groups all exhibited a transient decrease in body weight, which subsequently plateaued and then increased. This pattern has been observed in other ground-based models, as well as astronauts (Matsumoto et al., 2011; Stein 2012; Madokoro et al., 2018) (Figure 2C). During the experiment, the body weights of rats in both the control and sham groups significantly increased from baseline. In contrast, the body weights of rats in the simulated gravity groups did not significantly differ from baseline (Figures 2C,D). Considering the absence of substantial differences in cumulative food intake among the five groups, this phenomenon can be explained by reduction of musculoskeletal system weight in the three simulated gravity groups, which was influenced by the simulated reduction of gravity; importantly, it did not cause significant changes in body weight among the three simulated gravity groups.

In the present study, we did not observe significant decreases in the length and volume of hindlimb bones in the simulated gravity groups. Our findings differed from previous results (Ohira et al., 2003). Even though rats at this stage were experiencing a period of fast development, our rats were suspended 8 weeks after birth as opposed to 4 days after birth in the prior research. In addition, our rats were suspended for just 10 days, as opposed to about 3 months in the earlier study. We think the inability to detect significant changes in bone length and volume between the five experimental groups resulted from the shorter period of suspension and the older age of the rats.

In this experiment, we focused on changes in rat hindlimb bone parameters; we compared our findings with the results of previous studies to determine whether our NA could achieve a desired level of weight-bearing in rat hindlimbs. Our analyses of bone parameters in three different regions (proximal, middle, and distal) of femur and tibia revealed that the gravity reduction had significant effects on distal femur and proximal tibia near the knee joint (Figures 5D,H,L, 6B,F,J). These significant effects were mainly observed in the simulated gravity groups for overall BMD (Figures 5D, 6B), trabecular BMD (Figures 5H, 6F), MMA (Figure 7D,F), and PMA (Figure 7L,N). Also reflected in the cortical BMD is a portion of the simulated gravity group shown (Figures 5L, 6J).

Many studies have examined the relationship between the region-specificity of changes in BMD and exposure to different levels of gravity (Maupin et al., 2019; Tominari et al., 2019). Previous studies have shown that reductions in BMD are mainly concentrated in weight-bearing regions (LeBlanc et al., 2000; Stavnichuk et al., 2020). Our success in identifying a region-specific relationship between BMD alteration and gravity reduction implies that our NA can simulate low gravity in rats through a mechanism similar to the previous tail suspension apparatus that required ankle immobilization (Zhang et al., 2021).

Our experiment showed that distal femur and proximal tibia in the partial gravity groups did not exhibit a smaller degree of bone mass reduction, compared with 0*G*. Possible causes of these results include the inability of partial gravitational weight bearing (≤33%) to inhibit early osteocyte apoptosis; the persistent presence of partial gravity allows increases in osteoclast quantity and activity, which result in reduced bone growth and mass (Swift et al., 2013). In the future, we plan to use NA to establish additional partial gravity groups; we will investigate the potential for a linear relationship between the level of bone mass reduction and the level of gravity.

In the present study, we confirmed previously described differences in trabecular and cortical bone responses to gravity reduction: in distal femur and proximal tibia, trabecular BMD was much more sensitive than cortical BMD in the simulated gravity groups, which resulted in a significantly lower trabecular area percentage than the control group. Similar phenomena have been observed in rat models of hindlimb suspension, as well as astronauts (Bloomfield et al., 2002; Vico et al., 2017). Such results can be explained by the generally uniform distribution of cortical BMD relative to trabecular BMD across various partial weight-bearing groups, which suggests stable cortical bone responses after partial weight-bearing (Ko et al., 2020).

Cortical bone thickness is an essential consideration when assessing skeletal fractures. In the context of µ*G*-induced mechanical inactivity during spaceflight, the cortical endosteal surface expands outward, whereas the periosteal surface is stable or exhibits minimal movement; these factors lead to a decrease in cortical bone thickness (Gong and Zhang, 2010). Contrary to our expectations, we did not observe reductions of cortical bone thickness in distal femur or proximal tibia during exposure to low gravity. These findings might be explained by the absence of strong positional correlations between changes in cortical bone thickness and bone region. A previous study also failed to find a link between these alterations and the bones in specific regions (Maupin et al., 2019). In the future, we plan to explore the relationship between cortical bone thickness and skeletal location specificity by using larger numbers of experimental animals, extending the suspension period, and studying changes in the endosteum in different regions.

In some regions, our results differed from expectations: compared with the control group, bone parameters did not decrease in the µ*G* group, while they decreased in the Moon group (Figures 5L, 6A,N,P). These differences have been observed because the hindlimbs of the rats in the µ*G* group were affected by the 0*G* environment and other factors. Tail suspension in the µ*G* group caused a cephalad fluid shift because of the head-down tilt by approximately 30°, but rats in the Mars and Moon groups did not experience this effect. This fluid shift has been presumed to enhance perfusion of the cephalically situated skeleton, thus generating osteogenic stimulation; such stimulation may lead to the production of systemic anti-catabolic substances (Turner et al., 1994; Colleran et al., 2000). Anti-catabolic substances might have had a more pronounced effect on these sites, such that bone parameters were decreased in the Moon group but did not significantly change in the µ*G* group. The synergistic effect of tail suspension on rat hindlimbs in the µ*G* group should be further investigated in future studies.

Bone fragility and strength are important problems for human spaceflight. Astronauts must maintain healthy musculoskeletal tissues to perform critical and physically demanding missions. Thus, analyses of the mechanical indexes of various hindlimb regions during exposure to low gravity will provide essential guidance for astronauts during their missions in space and after they return to Earth. Two commonly used mechanical indexes, MMA and PMA, represent bone resistance to bending and twisting, respectively (Hisatomi and Kugino, 2019). In the present study, the regions that experienced the greatest adverse effects were also concentrated in distal femur and proximal tibia close to the knee joint (Figure 7D,F,L,N). Previous studies in humans and animals have revealed that force stimulation by weight-bearing activity increases bone MMA, thereby significantly increasing bone strength and reducing fracture risk (Warden et al., 2005, 2007; Kato et al., 2009). Our findings also confirm the previous reports that force stimulation is positively associated with PMA (Van der Meulen et al., 1995; Weatherholt et al., 2013). Additionally, we discovered significant reductions in both MMA and PMA in proximal tibia in the µ*G* group, compared with the Mars group. This phenomenon suggests that the increased gravity experienced by rats in the Mars group led to greater MMA and PMA in proximal tibia, compared with rats in the µ*G* group. These results indicate that our NA can be used to predict fracture risk while astronauts are on extraterrestrial objects.

The sham group in this study was established to determine whether the tail suspension would have additional effects on rats exposed to 100% hindlimb weight-bearing. We compared four bone parameters (overall BMD, trabecular BMD, MMA, and PMA in distal femur and proximal tibia) between the sham group and each of the three simulated gravity groups. We found that most simulated gravity groups showed significant decreases in these bone parameters, compared with the sham group. Thus, although rats in the sham group showed unique SA gnawing behavior, the presence of SA itself in the sham group did not have additional adverse effects on rat musculoskeletal health. Instead, low gravity was the leading cause of reductions in bone parameters among the three simulated gravity groups.

Our analysis of BMD, trabecular BMD, and cortical BMD in the five experimental groups confirmed that the reduction in gravity had a greater effect close to the knee joint; it also demonstrated that the mechanical indices MMA and PMA had the same characteristics. These results suggest that the NA was able to provide a stable and accurate partial gravity level in rat hindlimbs; thus, the original design purpose of the NA was achieved. Compared with previous devices, the NA has important advantages in terms of simulating partial gravity. First, the NA simulates partial gravity, which is determined by two factors: the DW of the rat and the weight of its balance container. The single daily assessment cuts down on the number of steps required for the experimenter and lessens the stress that too many measurements put on the rat. Second, The NA uses a flexible spring rope that allows the rat to have a range of motion within the cage similar to the control rats while ensuring that the hindlimbs are subjected to stable partial gravity. Using this spring rope in the NA reduces the risk of bone mass damage that a restricted range of motion may cause. Third, in this device, we did not use a suspension attachment to suspend the rat’s limbs, which would have ensured freedom of the rat’s joints and avoided additional bone loss due to the limited range of motion of the joints. Fourth, because the weight of the balance container is calculated by a formula and is accurate to one decimal place, the NA can be used to set a more accurate target weight level. Fifth, compared with our previous method that simulates µ*G* by immobilizing the rat’s ankle joint, the NA allows daily adjustment of balance container weight to ensure a more consistent and stable target level of gravity for the hindlimbs of an experimental rat.

Nevertheless, our NA experiment had some limitations. First, in the present study, we used tail suspension alone, rather than overall suspension. Tail suspension may gradually change the ratio of force on the four limbs of each rat; rats may begin to place more weight on their forelimbs, rather than their hindlimbs. In future long-term suspension experiments, we plan to refine the tail suspension, to overcome changes in force distribution in the forelimbs and hindlimbs. Second, this experiment analyzed the rat skeletal system without considering the muscular system; because muscles are sensitive to changes in gravity, the effects of the NA on muscle will be the mainly focus of future studies. Third, the micro-CT equipment we utilized was an early model, and the low scan resolution may be why we could not observe the decreased cortical bone thickness next to the knee joint. It also restricts our access to other bone parameters such as BV/TV, Tb. Th... We hope that new models of micro-CT devices will assist us in overcoming these objective issues in the future. Despite these limitations, we believe that the NA developed in this study offers a reliable simulator for studying the effects of changes in gravity on astronaut health during space missions; it may also be useful for studying the effects of gravity changes on the health of patients with various diseases who are bedridden and unable to support normal body weight. For researchers, the NA offers a promising new tool to study the alteration of gravity in space.

## Data Availability

The raw data supporting the conclusions of this article will be made available by the authors, without undue reservation.
