# Supplementary material for: A new type of simulated partial gravity apparatus for rats based on a pully-spring system
Source: Front Cell Dev Biol. 2022 Aug 31;10:965656. doi: 10.3389/fcell.2022.965656 (PMC9472129; doi:10.3389/fcell.2022.965656)
Supplement: Supplementary file 1 [file DataSheet2.pdf]

Suppl 2. Figure 1

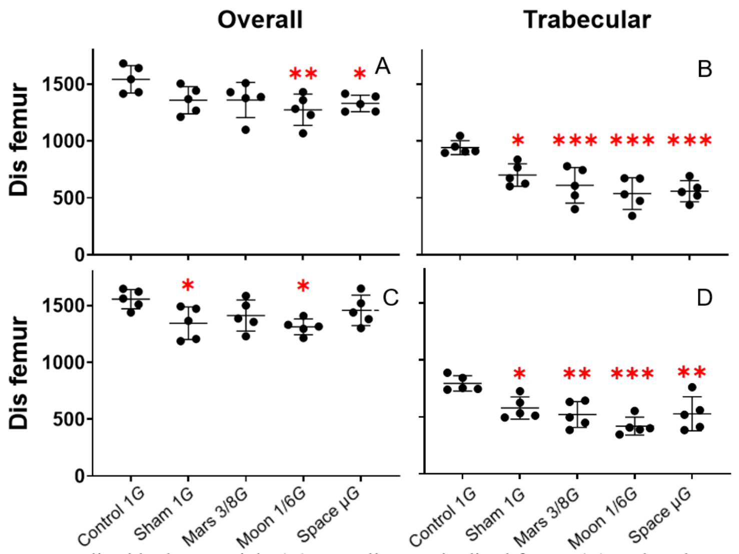

Suppl 2,Figure 1: BMD normalized by bone weight (**A**) Overall BMD in distal femur, (**B**) Trabecular BMD in distal femur, (**C**) Overall BMD in proximal tibia, and (**D**) Trabecular BMD in proximal tibia (Mean ± standard deviation, *p < 0.05 vs. control group,**p < 0.01 vs. control group, ***p < 0.001 vs. control group one- way analysis of variance).

Suppl 2. Figure 2

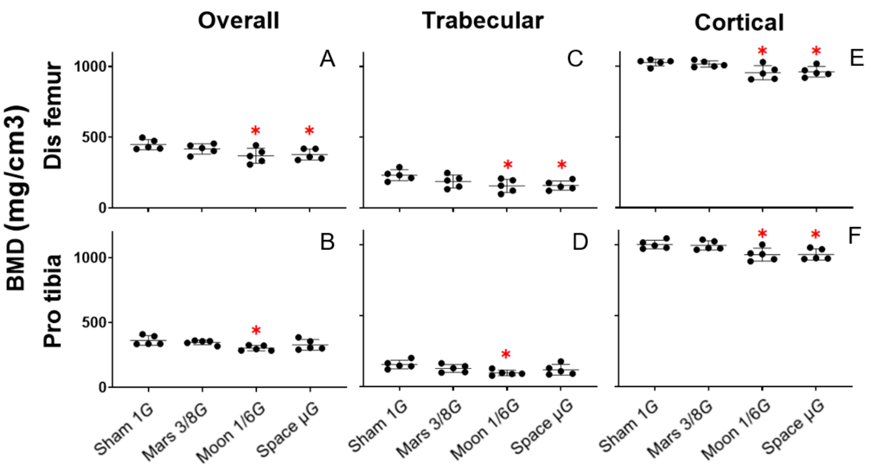

Suppl 2,Figure 2: Bone parameters of the distal femur and proximal tibia compared between the three simulated gravity groups and the sham group. (**A**) Overall BMD in distal femur, (**B**) Overall BMD in proximal tibia, (**C**) Trabecular BMD in distal femur, (**D**) Trabecular BMD in proximal tibia, (**E**) Cortical BMD in distal femur, and (**F**) Cortical BMD in proximal tibia (Mean ± standard deviation , one- way analysis of variance , *p < 0.05 vs. sham group).

Suppl 2. Figure 3

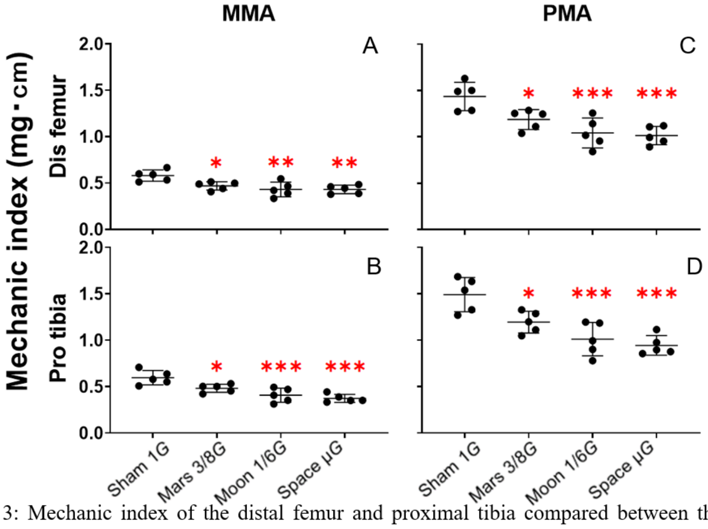

Suppl 2,Figure 3: Mechanic index of the distal femur and proximal tibia compared between the three simulated gravity groups and the sham group(**A**) MMA in distal femur, (**B**) PMA in distal femur, (**C**) MMA in proximal tibia, and (**D**) PMI in proximal tibia (Mean ± standard deviation, one- way analysis of variance , *$p < 0.05$ vs. sham group, **$p < 0.01$ vs. sham group, ***$p < 0.001$ vs. sham group).

Suppl 2. Figure 4

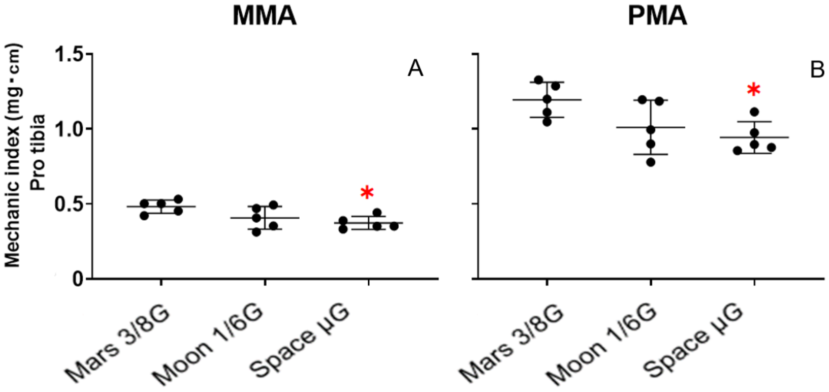

Suppl 2,Figure 4: Mechanic index compared among the three simulated gravity groups (**A**) MMA in proximal tibia, and (**B**) PMA in proximal tibia (Mean ± standard deviation, one- way analysis of variance *$p < 0.05$ vs. Mars group).
