# Supplementary material for: A new type of simulated partial gravity apparatus for rats based on a pully-spring system
Source: Front Cell Dev Biol. 2022 Aug 31;10:965656. doi: 10.3389/fcell.2022.965656 (PMC9472129; doi:10.3389/fcell.2022.965656)
Supplement: Supplementary file 2 [file Presentation1.PPTX]

## Slide 1
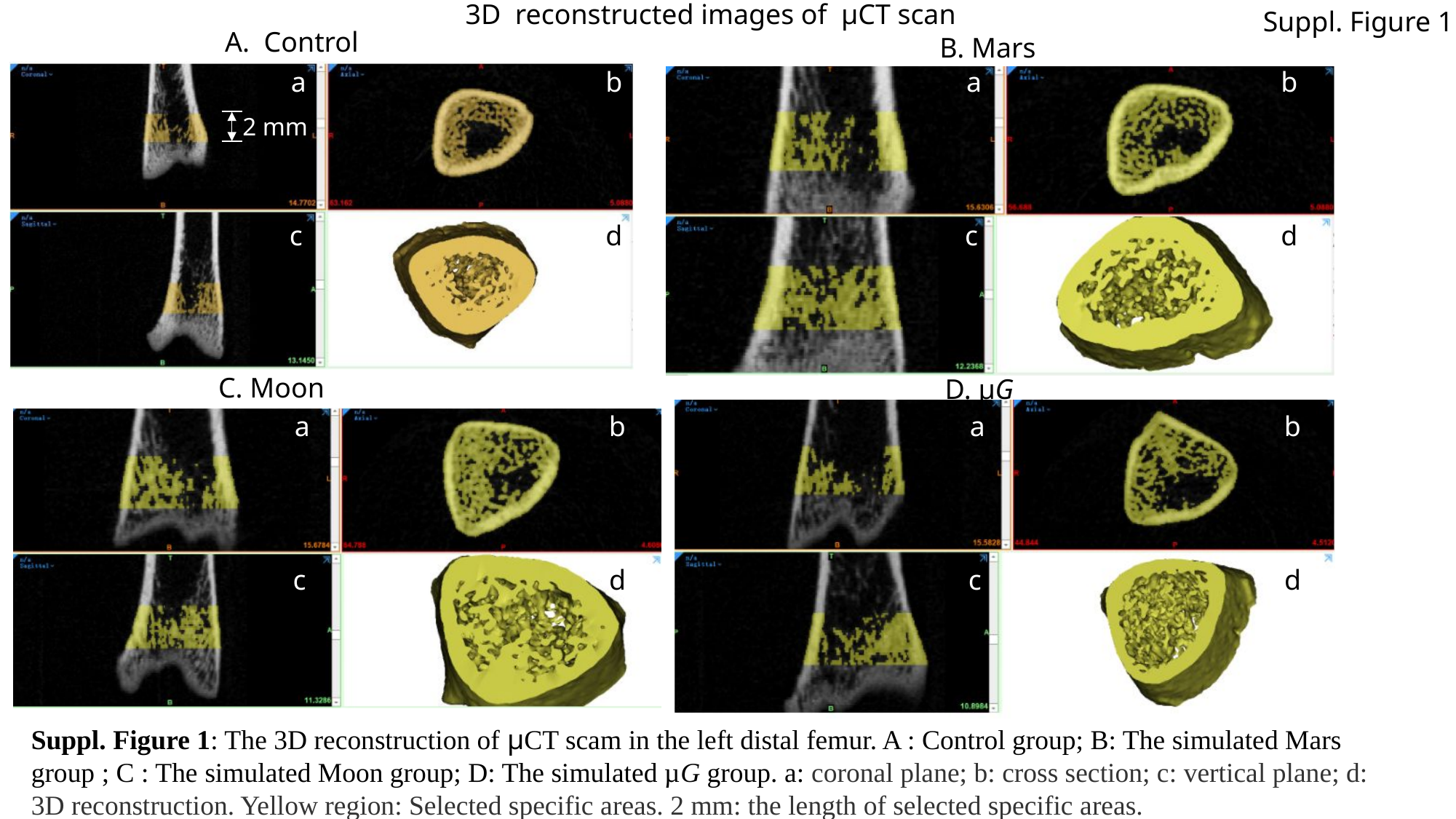

3D reconstructed images of µCT scan
Suppl. Figure 1
A. Control
B. Mars
a
b
c
d
a
b
c
d
2 mm
 C. Moon
D. µG
a
b
c
d
a
b
c
d
Suppl. Figure 1: The 3D reconstruction of µCT scam in the left distal femur. A : Control group; B: The simulated Mars group ; C : The simulated Moon group; D: The simulated µG group. a: coronal plane; b: cross section; c: vertical plane; d: 3D reconstruction. Yellow region: Selected specific areas. 2 mm: the length of selected specific areas.

## Slide 2
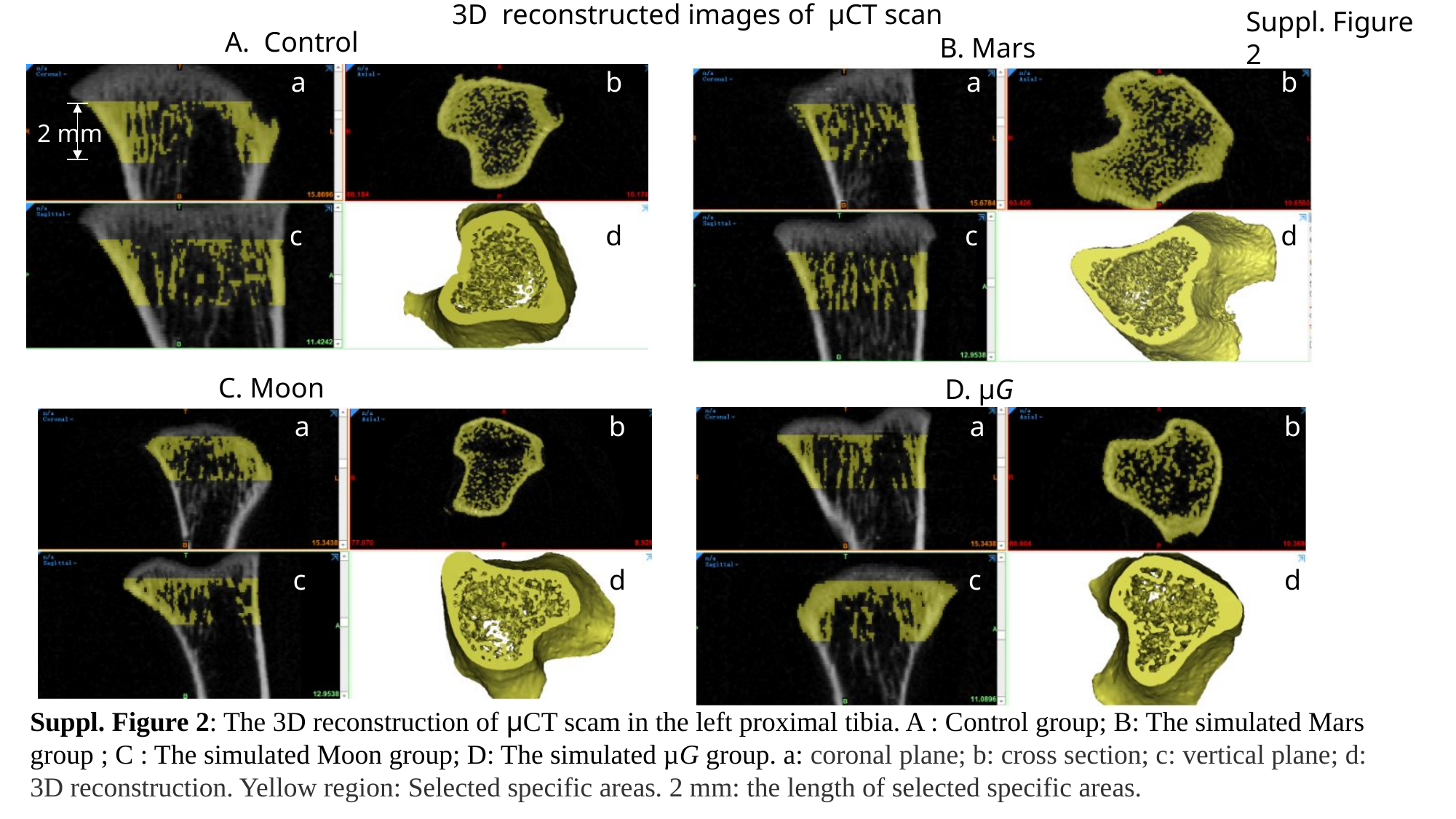

3D reconstructed images of µCT scan
Suppl. Figure 2
A. Control
B. Mars
a
b
c
d
a
b
c
d
 C. Moon
D. µG
a
b
c
d
a
b
c
d
2 mm
Suppl. Figure 2: The 3D reconstruction of µCT scam in the left proximal tibia. A : Control group; B: The simulated Mars group ; C : The simulated Moon group; D: The simulated µG group. a: coronal plane; b: cross section; c: vertical plane; d: 3D reconstruction. Yellow region: Selected specific areas. 2 mm: the length of selected specific areas.

## Slide 3
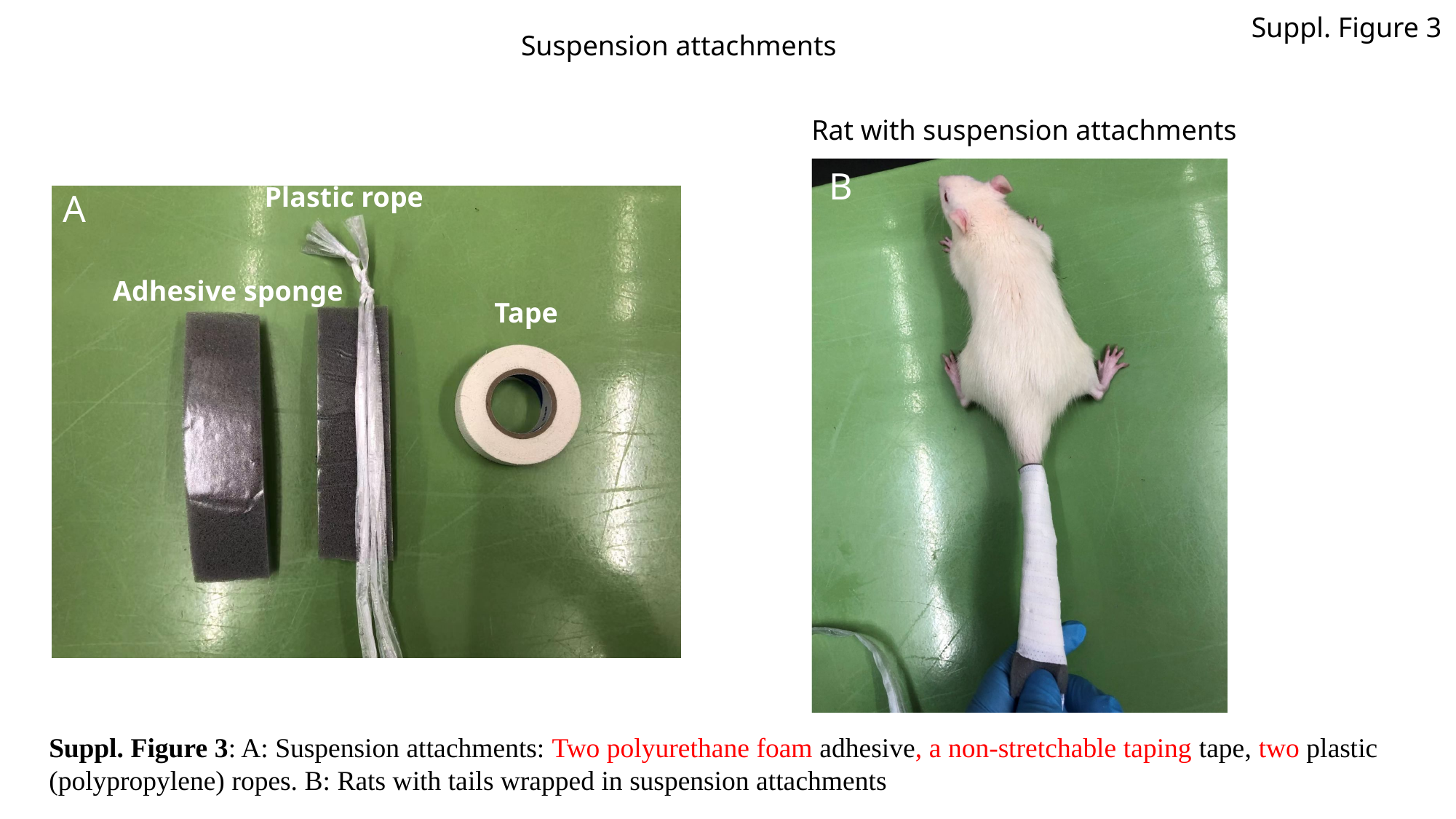

Suppl. Figure 3
Suspension attachments
Rat with suspension attachments
B
Plastic rope
A
Adhesive sponge
Tape
Suppl. Figure 3: A: Suspension attachments: Two polyurethane foam adhesive, a non-stretchable taping tape, two plastic (polypropylene) ropes. B: Rats with tails wrapped in suspension attachments

## Slide 4
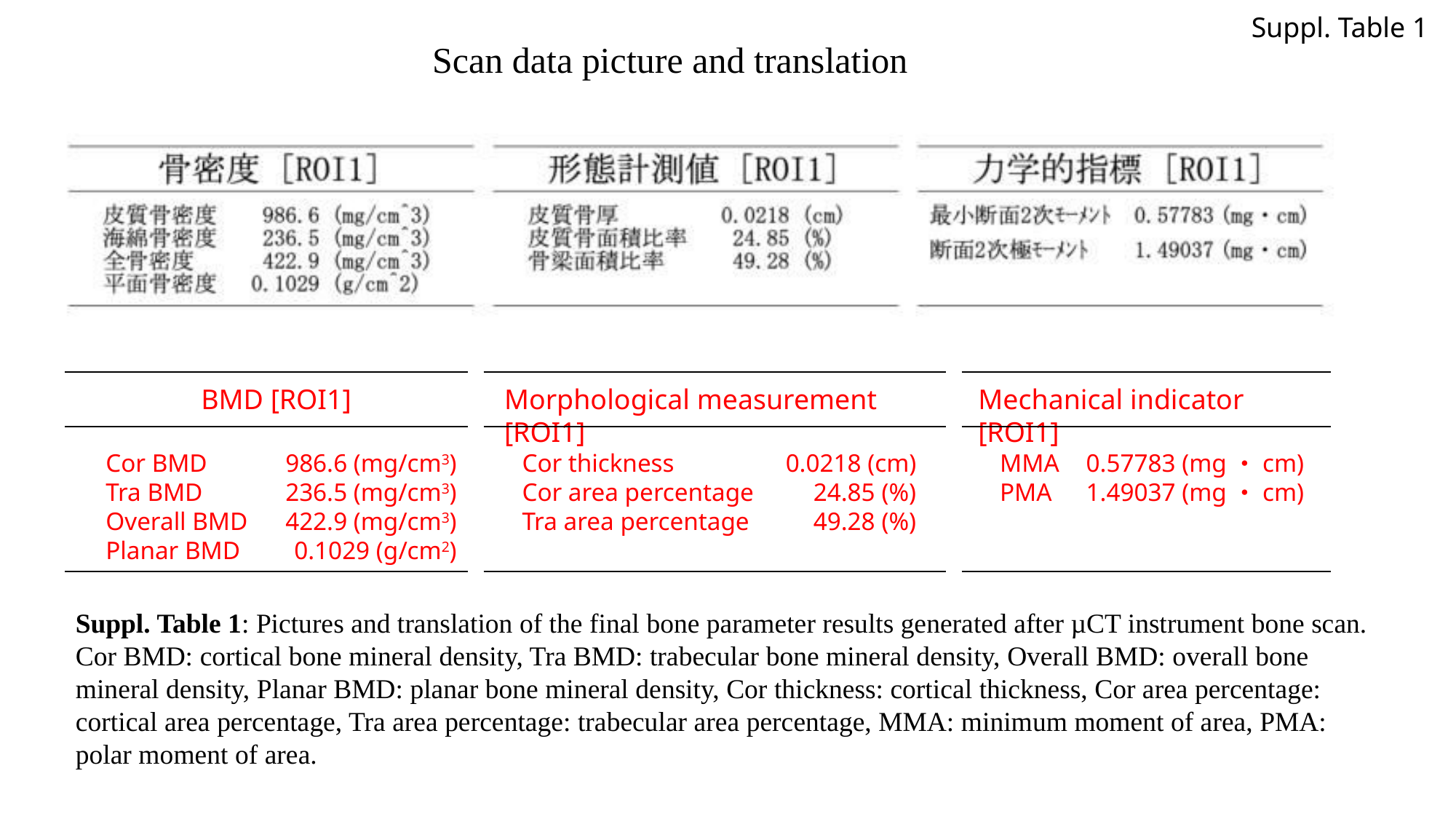

Suppl. Table 1
Scan data picture and translation
BMD [ROI1]
Morphological measurement [ROI1]
Mechanical indicator [ROI1]
Cor BMD
Tra BMD
Overall BMD
Planar BMD
986.6 (mg/cm3)
236.5 (mg/cm3)
422.9 (mg/cm3)
0.1029 (g/cm2)
Cor thickness
Cor area percentage
Tra area percentage
0.0218 (cm)
24.85 (%)
49.28 (%)
MMA
PMA
0.57783 (mg・cm)
1.49037 (mg・cm)
Suppl. Table 1: Pictures and translation of the final bone parameter results generated after µCT instrument bone scan. Cor BMD: cortical bone mineral density, Tra BMD: trabecular bone mineral density, Overall BMD: overall bone mineral density, Planar BMD: planar bone mineral density, Cor thickness: cortical thickness, Cor area percentage: cortical area percentage, Tra area percentage: trabecular area percentage, MMA: minimum moment of area, PMA: polar moment of area.
