# Supplementary figures and images for: A new type of simulated partial gravity apparatus for rats based on a pully-spring system
Source: Front Cell Dev Biol. 2022 Aug 31;10:965656. doi: 10.3389/fcell.2022.965656 (PMC9472129; doi:10.3389/fcell.2022.965656)

Control

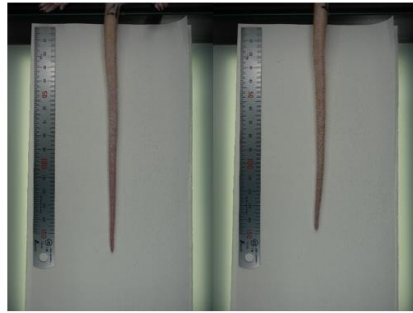

Simulated  $\mu$ G  
for 10 days

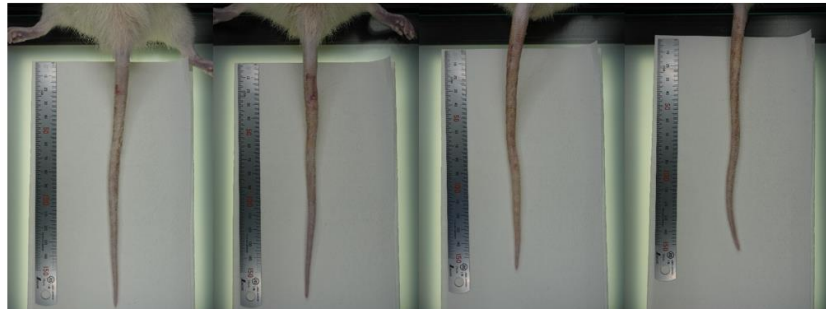

Supplement: Supplementary file 3 [file DataSheet1.pdf]
